# Integration of Sentinel-1A Radar and SMAP Radiometer for Soil Moisture Retrieval over Vegetated Areas

**DOI:** 10.3390/s24072217

**Published:** 2024-03-30

**Authors:** Saeed Arab, Greg Easson, Zahra Ghaffari

**Affiliations:** 1 Department of Geology & Geological Engineering, University of Mississippi, University, MS 38677, USA; sarab@contractor.usgs.gov; 2 Mississippi Mineral Resources Institute, University of Mississippi, University, MS 38677, USA; geasson@olemiss.edu; 3 Office of Research and Sponsored Programs, University of Mississippi, University, MS 38677, USA

**Keywords:** soil moisture, SMAP, Sentinel-1A, image fusion

## Abstract

NASA’s Soil Moisture Active Passive (SMAP) was originally designed to combine high-resolution active (radar) and coarse-resolution but highly sensitive passive (radiometer) L-band observations to achieve unprecedented spatial resolution and accuracy for soil moisture retrievals. However, shortly after SMAP was put into orbit, the radar component failed, and the high-resolution capability was lost. In this paper, the integration of an alternative radar sensor with the SMAP radiometer is proposed to enhance soil moisture retrieval capabilities over vegetated areas in the absence of the original high-resolution radar in the SMAP mission. ESA’s Sentinel-1A C-band radar was used in this study to enhance the spatial resolution of the SMAP L-band radiometer and to improve soil moisture retrieval accuracy. To achieve this purpose, we downscaled the 9 km radiometer data of the SMAP to 1 km utilizing the Smoothing Filter-based Intensity Modulation (SFIM) method. An Artificial Neural Network (ANN) was then trained to exploit the synergy between the Sentinel-1A radar, SMAP radiometer, and the in situ-measured soil moisture. An analysis of the data obtained for a plant growing season over the Mississippi Delta showed that the VH-polarized Sentinel-1A radar data can yield a coefficient of correlation of 0.81 and serve as a complimentary source to the SMAP radiometer for more accurate and enhanced soil moisture prediction over agricultural fields.

## 1. Introduction

The total amount of water in the unsaturated zone, known as soil moisture, plays a crucial role as a controlling factor in numerous hydrological processes [1,2,3]. It serves as a key variable in the examination of global water, energy, and carbon cycles. Additionally, soil moisture is a pivotal factor in watershed applications, including the monitoring of floods and droughts, the management of water resources, and the forecasting of crop yields [4]. Continuous soil moisture monitoring is crucial for assessing the risk of flooding and erosion, establishing sustainable irrigation policies, preventing wildfires, and many more applications [5,6]. In order to achieve more sustainable water consumption in agriculture, the systematic monitoring of available soil water content and its variations through space and time is needed.

Different methods have been established to measure soil moisture; in situ probes can measure soil moisture accurately and continuously at a single point, which means they can cover small areas and are not suitable for monitoring soil moisture for large areas over long period of time [3,5]. Another method involves proximal soil sensing techniques such as electromagnetic induction and ground-penetrating radar. They can cover larger areas than probes and provide accurate soil moisture data but with lower temporal resolution. Another method is remote sensing, including airborne and spaceborne techniques, which can provide global coverage and varying ranges of spatial and temporal resolution [3,5].

Several passive (SMMR, SSM/I, TMI, AMSR-E, and AMSR2) and active (ERS AMI and ASCAT) satellite-based sensors have collected global soil moisture data over the past few decades [7]. Statistical and physical synergic approaches were first introduced in NASA’s Soil Moisture Active Passive (SMAP) mission to benefit from the advantages of both active and passive sensors in order to achieve a higher resolution and more accurate measurement of soil moisture [8]. After the SMAP radar failed, the search for alternative sensors/approaches began to revive the mission’s high-resolution objective. The similar orbit configuration of the Sentinel-1A, relative to the SMAP radar, which allows for near-coincident observations, makes the Sentinel-1A radar the best candidate for integration with the SMAP radiometer. The main difference between the two sensors, which may affect the soil moisture remote sensing in dense vegetation conditions, is the difference in the frequencies of the SMAP L-band radiometer and Sentinel-1A C-band radar [9]. One other issue in the integration of SMAP and Sentinel-1A data is the coarse temporal resolution of the Sentinel-1A (12 days), which controls the temporal resolution of the synergic approach.

Combining Sentinel-1 and SMAP data could improve soil moisture estimates through downscaling methods [10]. Spatial downscaling involves converting a dataset with coarse spatial resolution into a more refined resolution. A significant amount of studies in the literature address the spatial downscaling of soil moisture data. According to [3], downscaling soil moisture falls into three main categories: 1—those utilizing geo-information data, 2—model-based approaches, and 3—satellite-based methods. Geo-information-based methods establish connections between coarse-scale soil moisture data, geological attributes, and fine-scale soil moisture values. Model-based methods can be divided into two categories: statistical models and land surface models. Statistical models use the insights derived from research on the spatial statistics of soil moisture and the variations in statistics across different scales. In the land surface model, course-scale observations are used to optimize the hydrological or land surface model parameters. And statistical regression is applied to integrate coarse-scale observations in land surface models [1,3,11]. Satellite-based methods fall into two categories: Firstly, there are active and passive microwave data fusion methods, which have been widely exploited to estimate soil moisture during the past few decades. Passive microwave radiometers have higher temporal resolution but coarse spatial resolution. On the other hand, active microwave sensors can provide higher spatial resolution but have coarse temporal resolution. Secondly, optical and microwave fusion methods are used to enhance the spatial resolution of soil moisture data. More details on each downscaling method can be found in the review paper by the authors of [3].

Ref. [12] used SMAP’s L-band Synthetic Aperture Radar (SAR) backscatter measurements at high resolution (1–3 km) to downscale SMAP L-band brightness temperature measurements at coarse resolution (~40 km) to a 9 km brightness temperature. The downscaling algorithm is based on the correlation between temporal fluctuations in brightness temperature and backscatter, particularly evident when observing targets simultaneously at identical angles. Their results show that the incorporation of SAR measurements can enhance the accuracy of high-resolution soil moisture retrieval. In an airborne field experiment, the error was diminished by 40%, falling within the tolerance specified by the SMAP data product requirements.

Ref. [10] combined Sentinel-1 and SMAP data to improve soil moisture estimates. In their process of assimilation satellite observations, they directly ingested Level-1 observations in the three-dimensional Ensemble Kalman Filter (3-D EnKF) model. Their results indicated an enhanced spatiotemporal accuracy for the soil moisture estimates. The assimilation of SMAP and Sentinel-1 data yielded the best results, highlighting the complementary value of radar and radiometer observations.

In vegetated areas, the high spatial resolution advantage of radar systems for soil moisture retrieval is mitigated by the higher sensitivity of radar to vegetation scattering [13]. Scattering from the vegetated areas incorporates the volume scattering from the vegetation cover and surface scattering from the underlying soil [14]. Incorporating optical remote sensing-derived land surface parameters such as surface temperature and the Vegetation Index into the synergy model can significantly improve the accuracy of soil moisture retrieval over the vegetated area. In this study, the surface temperature and Vegetation Index data of the Visible Infrared Imaging Radiometer Suite (VIIRS) were included in the synergy model to account for the vegetation scattering of the radar and to improve the retrieval algorithm [15].

The coarse spatial resolution of SMAP limits its potential for local-scale soil moisture monitoring. The purpose of this study is to address the loss of the high-resolution capability in the SMAP mission due to the failure of the radar component. In this paper we propose a relatively simple algorithm for enhancing the spatial resolution of the SMAP up to the resolution of Sentinel-1A. In this study we utilized the Smoothing Filter-based Intensity Modulation (SFIM) method to downscale the radiometer data. The algorithm relies on a multi-sensor image fusion method, which is usually employed with multispectral images and panchromatic data. Then, we employed an Artificial Neural Network (ANN) to exploit the synergy between the Sentinel-1A radar, SMAP radiometer, and in situ-measured soil moisture. The analyses focus on the plant growing season of 2016 over the Mississippi Delta region.

## 2. Materials and Methods

### 2.1. Study Area and Data

#### 2.1.1. Study Area

The Mississippi Delta was our study area. The Mississippi Delta is an important surficial aquifer and is located in the northwest of Mississippi, with an area of 18,100 km^2^. This area is covered with dense agricultural activities. Because of its fertile soil, more than 50% of the Mississippi Delta region is cultivated. The majority of the Mississippi Delta is covered by a thick layer of silt–clay, which makes it an impermeable layer in most parts. Because of this, not much of the 1300 mm of annual rainfall in the area can be absorbed. Despite this amount of rainfall and the presence of the Mississippi river in the area, groundwater resources face stress. This is due to the utilization of over 90% of its supply for irrigation, leading to significant declines in water levels in certain areas of the aquifer. Soybean, cotton, corn, and rice are the dominant types of crops in the region [16,17]. Field data collection was performed during the growing season (April to August) of 2016 to measure the soil moisture and assess the accuracy of soil moisture remote sensing systems.

#### 2.1.2. Field Data

**Figure 1:**
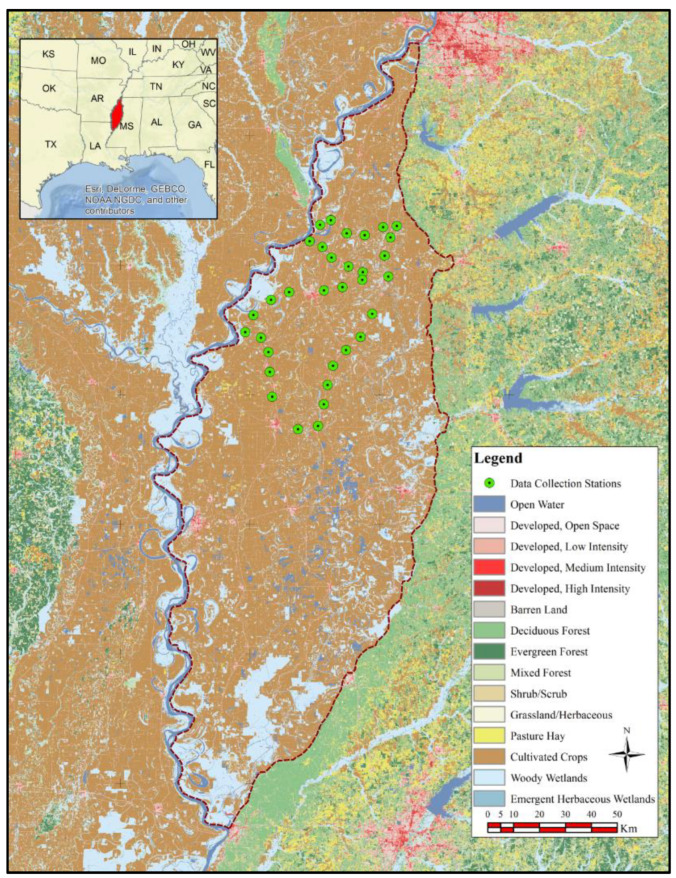
Study area and 33 collection stations inside the study area [18].

We collected soil moisture data in five counties in the Mississippi Delta during the plant growing season. The distribution of the measurement stations that we visited monthly to capture soil moisture data is illustrated in Figure 1. Each study day involved the collection of soil moisture data at 33 stations using a soil moisture probe [18]. At each station, data were gathered at various nearby locations, ensuring the representation of the heterogeneity in both land surface and soil moisture with respect to satellite ground sampling distance. The consistency in soil type, elevation, and land cover across the visited fields ensures that in-situ observations are representative on the scale of satellite pixels. The collected data were then averaged to provide a comprehensive picture of the soil moisture conditions.

For this study, we used Waterscout™ SM100 and the Fieldscout^®^ (Spectrum Technologies, Inc., Plainfield, IL, USA) soil sensor reader to measure volumetric soil moisture information instantaneously. The sensor is designed to provide a nominal accuracy of 3% in measuring soil moisture under conditions where the soil’s electrical conductivity is less than 8 MilliSiemens/cm, a typical electrical conductivity range in agricultural fields [19]. Moreover, in locations where the soil was approaching its plastic limit, a soil sample was gathered for calibration purposes. The soil moisture content of these samples was determined in the laboratory using the gravimetric method outlined in the ASTM D 2216-98 instructions [20].

#### 2.1.3. Sentinel-1A Level 1 GRD

Sentinel-1A (2014-present) is a Synthetic Aperture Radar (SAR) sensor that provides C-band observations at fine spatial resolution. Interferometric Wide (IW) swath mode has a nominal spatial resolution of 5 × 20 m over 250 km swath, with a global revisit time of 12 days. The Level-1 Ground Range Detected (GRD) product is formed through the processing of focused SAR data. These data are initially detected, multi-looked, and then projected onto the ground range using an Earth ellipsoid model. The ellipsoid projection of GRD products undergoes correction based on terrain height information specified in the product’s general annotation. While the terrain height varies in azimuth, it remains constant in the range direction. Ground range coordinates represent the slant range coordinates projected onto the Earth’s ellipsoid. The pixel values in the resulting product signifies detected magnitudes. The product features approximately square resolution pixels (20 × 22 m) and uniform pixel spacing (10 × 10 m), aiming to minimize speckles at the expense of reduced resolution.

In this study, the Level 1 GRD data with 10 m pixel spacing was selected for high-resolution soil moisture retrieval during the growing season. Data were acquired from ASF DAAC [21]. The radiometric calibration, speckle filtering, and geometric correction procedures were applied to the SAR intensity data, and the backscatter coefficient values [*σ*^0^(dB)] were calculated.

#### 2.1.4. SMAP L3 Brightness Temperature

The SMAP was launched in 2015, and it was the first soil moisture observation mission designed to integrate an L-band SAR instrument (active microwave instrument operating at 1.22–1.3 GHz) and an L-band radiometer (passive microwave operating at 1.41 GHz) functioning concurrently. Although the radar experienced a malfunction shortly after the mission’s commencement, the radiometer remains operational. The SMAP has a unique technical capability to detect and mitigate radio frequency interference [22].

The passive radiometer of the SMAP captures brightness temperature measurements in both vertical and horizontal polarizations, along with the third and fourth Stokes parameters. These measurements are then mapped onto a 36 km resolution Equal-Area Scalable Earth (EASE-2) grid [15] through inverse distance gridding [22,23,24]. The science data available at the NSIDC DAAC are interpolated and posted to the EASE-Grid. In this study, the 9 km level 3 horizontally polarized brightness temperature (SPL3BT) data [25] were used to exploit the synergy model for soil moisture retrieval.

#### 2.1.5. VIIRS Land Surface and Vegetation Index Data

The Visible Infrared Imaging Radiometer Suite (VIIRS) sensor is a scanning radiometer onboard the Suomi NPP, NOAA-20, and NOAA-21 satellite series, which collects visible and infrared imagery and radiometric measurements of land, the atmosphere, the cryosphere, and oceans. The VIIRS instrument was created to combine and, whenever feasible, enhance the most advantageous features of the Moderate-resolution Imaging Spectroradiometer (MODIS), the Advanced Very-High-Resolution Radiometer (AVHRR), the Sea-viewing Wide Field-of-view Sensor (SeaWIFS), and the Operational Linescan System (OLS). VIIRS data are distributed in two main formats: Sensor Data Records (SDRs), categorized as Level-1 products, and Environmental Data Records (EDRs), classified as Level-2 products, according to NASA’s terminology [26]. In this research study, we used Level-1 data. The VIIRS Land Surface Temperature (LST) data collected after April 2014 and Vegetation Index data collected after August 2014 were validated and are of good quality for science use. The spatial resolution of the LST product is 0.75 km at nadir. The VIIRS Vegetation Index (VI) product is generated daily at the resolution of 0.375 km at nadir over land in swath form. VIIRS data are archived and distributed by the NOAA Comprehensive Large Array-data Stewardship System (CLASS) [27]. The Top of Atmosphere–Normalized Difference Vegetation Index (TOA-NDVI) and LST data that are collected during the ascending node (crossing the equator around 13:30 local time) were used in this study.

Before using remote sensing data for analysis, the data must be coregistered. Given the 1 km target resolution for the final soil moisture product, the finer-resolution VIIRS and Sentinel-1A data were aggregated to 1 km using an averaging filter, and the coarser SMAP data were disaggregated to 1 km using the downscaling method described in Section 2.2.1.

### 2.2. Methods

**Figure 2:**
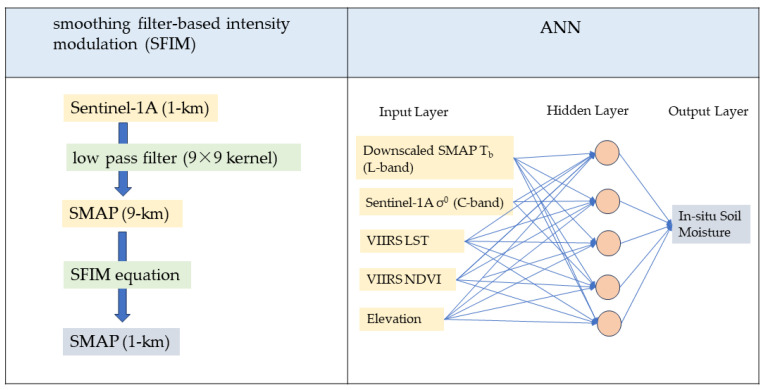
Schematic approaches for downscaling the SMAP soil moisture data.

In this research study, the SMAP soil moisture data were downscaled to a finer resolution in two steps. First, we used a dynamic image fusion to downscale the spatial resolution of the image. Second, we used a synergic method, specifically an Artificial Neural Network (ANN) method, to downscale the soil moisture data. The downscaling approaches are shown in Figure 2.

#### 2.2.1. Spatial Resolution Downscaling

The downscaling method used in this study uses a dynamic image fusion and is derived from the Smoothing Filter-based Intensity Modulation (SFIM) technique [28]. SFIM is a spectral preservation fusion technique used to enhance spatial resolution [29], and it is usually applied to multispectral images and panchromatic data [28]. The main assumption in using this technique in this study was that the backscatter coefficient (*σ*^0^) of the Sentinel-1A SAR sensor is correlated with the brightness temperature (*T_b_*) of the SMAP radiometer.

The downscaling algorithm consists of two steps. In the first step, a low-pass filter is used to degrade the resolution of the Sentinel-1A SAR data to the resolution of SMAP radiometer product. The low-pass filtering calculates the local mean and removes noise by reducing the significance of anomalous pixels. The filtering kernel size is decided based on the ratio between the coarse- and fine-resolution data. The kernel used to degrade a 1 km Sentinel-1A product to 9 km is 9 × 9, defined as follows:

(1) $$H\left(u,v\right)=\frac{1}{81}{\left[\begin{array}{ccc} 1 & \begin{array}{cc} 1 & \cdots \end{array} & 1\\ \begin{array}{c} 1\\ \vdots \end{array} & \ddots & \begin{array}{c} 1\\ \vdots \end{array}\\ 1 & \begin{array}{cc} 1 & \cdots \end{array} & 1 \end{array}\right]}_{9\times 9},$$

**Figure 3:**
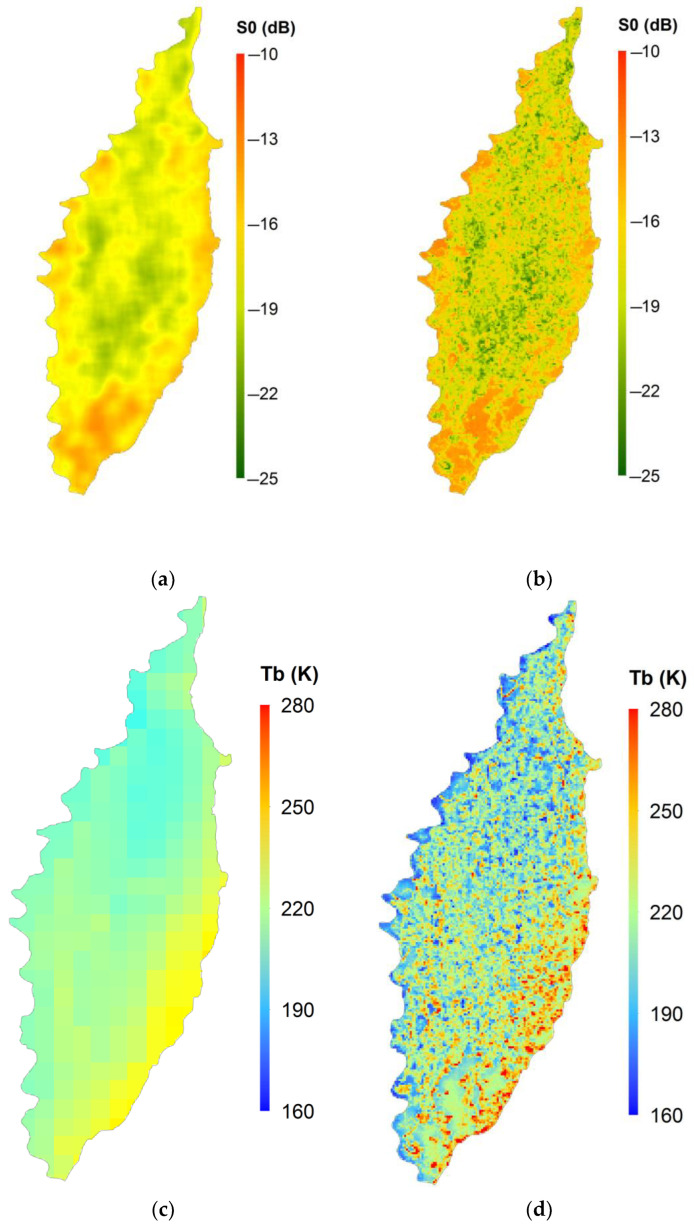
SFIM downscaling. (**a**) Aggregated 1 km SAR data, (**b**) low-pass-filtered SAR data, (**c**) 9 km SMAP brightness temperature, and (**d**) downscaled brightness temperature.

In the second step, the SMAP *T_b_* is downscaled to SAR resolution using the ratio between the original low-resolution *T_b_* and degraded (low-pass-filtered) *σ*^0^ in the SFIM equation.

(2) $$T_{b\_Hres}=\frac{T_{b\_Orig}}{{\sigma^{0}}_{LPF}}\times {\sigma^{0}}_{Orig},$$

where *T_b_Hres_* is the downscaled brightness temperature, *σ*^0^_*LPF*_ is the degraded *σ*^0^, *T_b_Orig_* is the 9 km T_b_ of the SMAP, and *σ*^0^_*Orig*_ is the aggregated 1 km *σ*^0^ of the Sentinel-1A. Figure 3a shows the aggregated 1 km SAR data, while the data that were degraded with the low-pass filter are shown in Figure 3b. Figure 3c shows the 9 km SMAP *T_b_*, which is downscaled to 1 km resolution, as shown in Figure 3d.

#### 2.2.2. Synergic Approach

Among different network structures, feed forward back propagation has been proven as an efficient architecture for detecting complex relationships between variables in remotely sensed data. The ANN method used in this study is a feed-forward Multi-Layer Perceptron (MLP) with a few neurons in the hidden layer. ANNs demonstrate versatility, effectiveness in modeling non-linear systems, and precision and are easy to use [30]. In this type of ANN model, an algorithm was utilized to compute the output error in the forward propagation phase. Following this, the error was allocated to each weight during the backward propagation process [31]. The number of hidden nodes was ascertained through a trial-and-error approach, wherein different configurations were tested to optimize the model’s performance. The input layer consisted of SMAP *T_b_*, Sentinel-1A *σ*^0^, and VIIRS surface temperature and Vegetation Index data, and the output layer was the in situ-measured soil moisture data. Out of the total ~100 data points collected over three study dates, 70% of the data were used for training, 15% for validation, and 15% for testing the network. The validation data were used for network generalization and to halt training when the generalization stopped improving. The testing data had no effect on training and provided an independent measure of network performance during and after training. The network was trained using the Levenberg–Marquardt backpropagation algorithm.

## 3. Results

**Figure 4:**
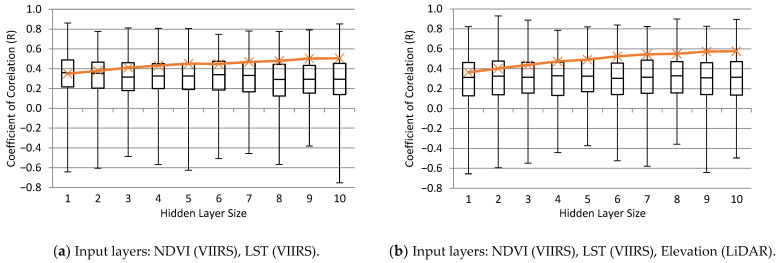

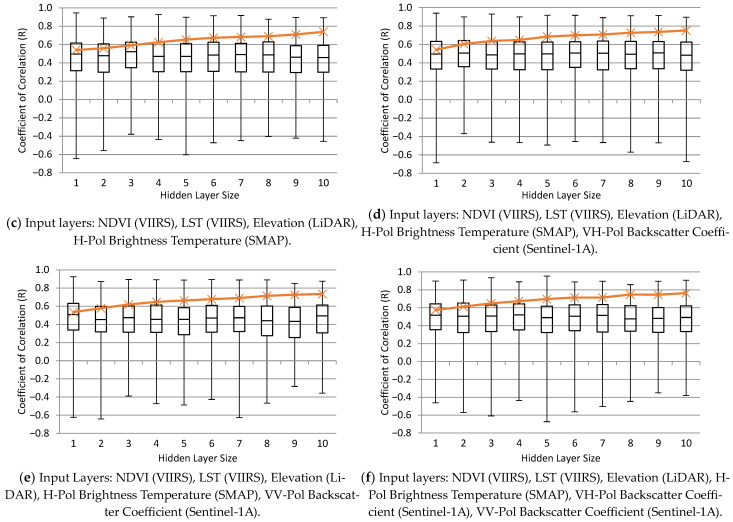
Performance results for different neural network structures and input layers.

Neural network learning was performed for six additive sets of input data. The parameters in the input layer of the neural network are shown in Figure 4. The red line shows the change in the mean coefficient of correlation (R) for the training set over the 500 runs as the hidden layer size (number of neurons in the hidden layer) was changed. The box and whisker plots show how the test accuracy varied in the 500 runs at a constant hidden layer size. The best performance that the synergic approach can achieve with all land surface parameters occurs when more than 50% of the runs have an R > 0.5. This proves the complex nature of soil moisture retrieval over an agricultural setting when the crops have matured (August and September). Another important reason is the relatively small size of the training dataset (75 points), which could lead to weak training in half of the runs. The standard deviations of the testing set accuracy in 500 runs were also high, which indicates that the network performance significantly depends on the selected training set.

**Figure 5:**
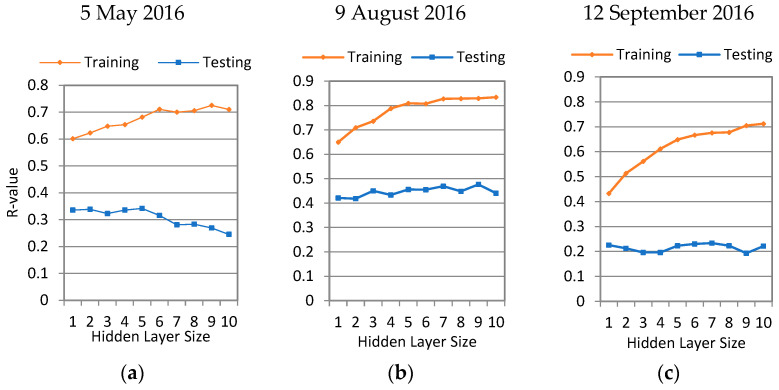
The neural network performance results for (**a**) 5 May 2016, (**b**) 9 August 2016, and (**c**) 12 September 2016 study dates.

If we consider a neural network with R > 0.8 as a very good model [32], a small percentage of the neural networks created using all the data from May, August, and September meet that criterion. In the next step, the network training was repeated separately for each study date to uncover the effect of growing vegetation. The network performance results for each study date are shown in Figure 5. As the figure suggests, the average R-value of the testing set increases from May to August and decreases in September. Most of the fields in the Mississippi delta are covered with green crops, and the landcover (NDVI value) is more homogenous in early August, and therefore, neural network predictions are more reliable. In early May and mid-September, there is more heterogeneity in the NDVI values, which leads to a weaker performance for the neural network. The standard deviation values drastically increased for all three dates, which highlights the importance of the training set size (in each date, there are 23 data points in the training set and 5 data points in each of the validation and testing sets).

Figure 5 also suggests that the networks tend to overfit as the hidden size layer increases. The average R-value of the training set improves with more neurons, as expected, but the average R-value of the testing set remains stable or, in some cases, decreases (Figure 5a,c).

The neural network analyses indicate that (1) only one neuron in the hidden layer would be optimal for the hidden layer size and that (2) relative elevation (and, consequently, soil type) does not have significant effect on the network performance. This affirms that the elevation and soil type variations in the Mississippi Delta are very minimal. (3) The most significant improvement in the network performance was made by adding the SMAP L-band Tb in the input layer which improved the average R-value of the testing set from 0.3 to 0.45. (4) The Sentinel-1A C-band VV-pol *σ*^0^ did not improve the average R-value of the testing set, but there were slights improvements from the VH-pol *σ*^0^.

Table 1 shows the good regression results of the network training for the input Sentinel-1A data scenarios, including Sentinel-1A VH polarized *σ*^0^, VV polarized *σ*^0^, and both dual-polarization modes’ data. At a constant hidden layer size, the network with VH-polarized *σ*^0^ in the input layer results in better regression parameters for the testing dataset in most of the attempts.

**Figure 6:**
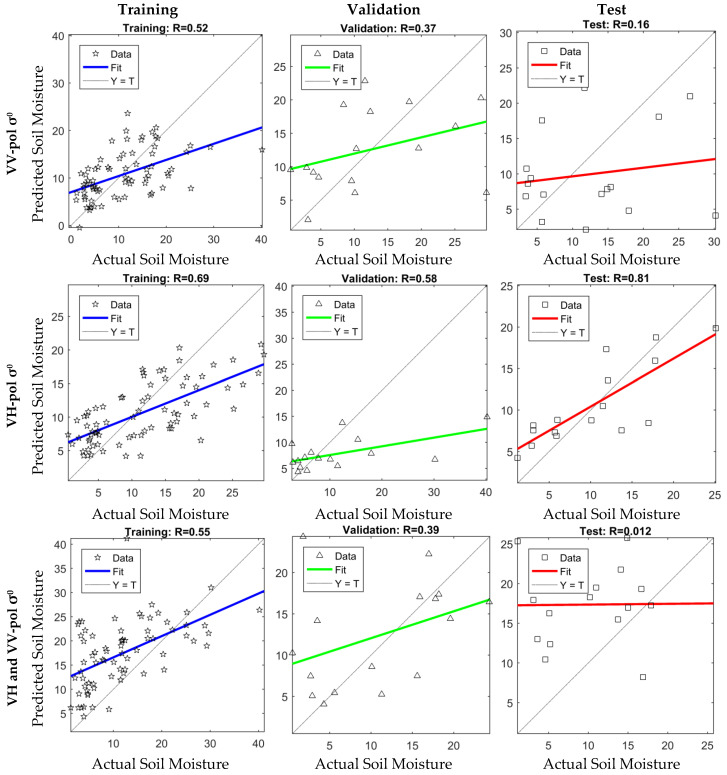
The neural network training results.

Detailed network training results are shown in Figure 6. The in situ-measured soil moisture is displayed on the *x*-axis, and the *y*-axis shows the predicted soil moisture from the neural network. The coefficients of correlation for the training, validation, and test datasets are displayed at the top of each panel.

**Figure 7:**
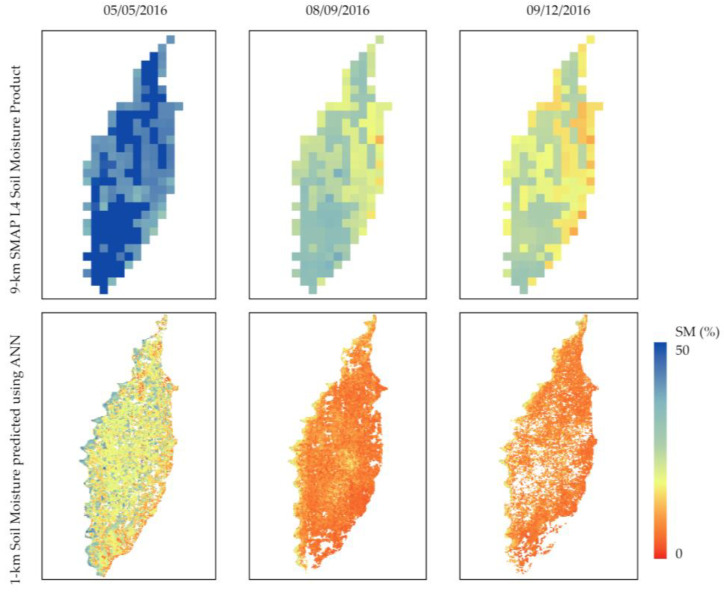
Soil moisture maps created using the neural network.

After the training was complete, the network with an R-value of 0.81 was used to estimate soil moisture for the study site. The resulting soil moisture maps are illustrated in the bottom panels of Figure 7. The SMAP 9 km L4 Soil Moisture product is also displayed as a reference to show the improved spatial resolution using the synergy approach. The higher soil moisture content along the Mississippi River is captured in the downscaled maps. The no-data pixels in the downscaled soil moisture maps are due to cloud contamination in surface temperature and VI data from VIIRS. The lower soil moisture values in the downscaled maps compared to the original SMAP product for the studied days indicate that the SMAP is overestimating the soil moisture. SMAP overestimation is attributed to multiple factors, including the error in vegetation water content estimation. Ref. [33] observed that during the period of high NDVI values, the SMAP algorithm overestimates the soil moisture. Ref. [34] found that SMAP measurements are systematically higher than in situ soil moisture values. Other studies of the SMAP soil moisture [35,36] have indicated soil moisture underestimation in vegetated conditions, but the accuracy of the in situ-measured soil moisture data is a key factor in validation results. For instance, the Soil Climate Analysis Network (SCAN) sites used in [35] were reporting biased values in the Mississippi Delta during the growing season of 2016 due to high vegetation density over the site and, therefore, were not used in our study. We believe downscaled soil moisture maps provide more accurate estimations of soil moisture. The soil moisture values in the downscaled maps are consistent with the results reported in [18], which studied the same geographic region on the same observation dates.

## 4. Discussion

The remote sensing of soil moisture over agricultural fields during the plant growing season is complicated because vegetation and surface roughness reduces the sensitivity of microwave observations to soil moisture [37,38], and as the vegetation density and water content increases, the accuracy of soil moisture retrieval systems using microwave remote sensing significantly decreases. The SMAP was designed as a combination of L-band active and passive sensors and is currently operating with the passive radiometer only, which means it does not achieve the desired accuracy over agricultural settings. The radiometer-derived soil moisture is overestimated over the cropland areas due to the soil effective roughness and vegetation [39]. According to [39], the accuracy over croplands were the worst, and the SMAP values were overestimated in wet areas. In contrast, ref. [40] evaluates SMAP/Sentinel-1 products and confirms the high correlation between SMAP/Sentinel-1 and in situ measurements (R-values between 0.19 and 0.95). Ref. [40] confirmed that the accuracy of SMAP/Sentinel-1 products is not dependent on water bodies, urban areas, and soil types. In that study, SMAP/Sentinel-1 products were found to have a high correlation with grassland and cropland reference data due to the application of vegetation effect correction in the active–passive algorithm. Different radar datasets and synergy methods have been tested to enhance the accuracy of the SMAP radiometer [41,42,43], but few have studied dense vegetation conditions. The ANN approach used in this study provides a powerful tool for estimating soil moisture in vegetated areas by leveraging the SMAP radiometer and identifying its relationship with active microwave data, as well as ancillary surface parameters.

Ref. [44] implemented the Backus–Gilbert (BG) optimal operation algorithm to improve the Level-1C Enhanced Brightness Temperature (L1C_TB_E) product to provide an optimal interpolation of the radiometer measurements onto a global 9 km grid. They concluded that the SMAP sampling pattern results in overlapping measurements which, together with BG optimal interpolation, result in a more accurate estimation of brightness temperature. The findings reported by the authors of [12], who downscaled SMAP data from ~40 km to 9 km spatial resolution, demonstrate that the suggested baseline, which integrates both active and passive components of the SMAP mission, is sufficient for meeting its Level-1 requirements and adheres to the specified error budget across various regions worldwide.

Also, ref. [10] combined Sentinel-1 and SMAP data to improve the soil moisture data from ~40 km to 9 km. They concluded that incorporating Sentinel-1 data enhances the assimilation impact by up to 30% compared to the impact observed with SMAP-only assimilation. Similar to the authors of [10,12], we increased the spatial resolution of soil moisture data by integrating the Sentinel-1A Radar and SMAP.

In our analysis, using one neuron in the hidden layer was sufficient, and networks tended to overfit as the hidden layer size increased. Relative elevation (and consequently soil type) did not have a significant effect on the network performance. This is likely due to minimal variations in the elevation and soil type in the Mississippi Delta. Among the training datasets, the most significant improvement in the network performance was made by adding the SMAP L-band *T_b_* in the input layer, which improved the average R-value of the testing set from 0.3 to 0.45. The Sentinel-1A C-band VV-pol *σ*^0^ did not improve the average R-value of the testing set. The average R-value of the testing set increased from May to August and decreased in September. The standard deviation of the testing set accuracy in 500 runs for all three dates was relatively large.

## 5. Conclusions

This study addresses the challenge posed by the failure of NASA’s SMAP radar component by proposing and evaluating a solution that integrates an alternative radar sensor, ESA’s Sentinel-1A C-band radar, with the SMAP radiometer. The novelty of our study stems from (i) accurately measured and well-calibrated soil moisture data in a vegetated area, (ii) the downscaling approach, which can be generalized to estimate soil moisture at sub-kilometer to medium (10–100 m) resolution by simply using finer-resolution surface ancillary data; and (iii) the use of vegetation-describing surface parameters derived from optical remote sensing to enhance the retrieval accuracy of soil moisture over a densely vegetated area.

The synergic approach proposed in this paper provides a viable method for taking advantage of available radar data and improving the spatial resolution of SMAP radiometer soil moisture products to sub-kilometer and even finer spatial resolutions. The main challenge in achieving such fine spatial resolution over vegetated areas is the availability of ancillary land surface data. Our results suggest that using the Sentinel-1A VH-polarized backscatter coefficient improves the SMAP’s performance over vegetated areas. The C-band radar used in this study could provide more significant improvements in soil moisture estimation over less vegetated soil conditions. For densely vegetated areas such as the Mississippi delta (studied in this manuscript), an L-band radar is expected to yield more accurate soil moisture estimations. It is important to emphasize that a good training dataset with a sufficient sample size, measurement accuracy, and density can significantly improve the ANN’s performance to fully exploit the multi-sensor and multi-modal observations of soil moisture signals.

This research provides a potential pathway for advancing our capabilities in soil moisture retrieval with finer spatial resolution, which will ultimately serve as a key variable for environmental monitoring, water management in agriculture, wildfire management, and other applications. Future research in this area could lead to more accurate soil moisture estimations with finer spatial resolution. In particular, leveraging the L-band radar observations and using medium-resolution ancillary surface parameters from Landsat and Sentinel-2 can improve downscaling results.

## Figures and Tables

**Table 1:** Regression results for three different neural networks.

Input Layer	Output Layer	Testing Results	
		Root Mean Squared Error (m^3^/m^3^)	Regression R-Value
NDVI (VIIRS)	Soil Moisture	3.97	0.81
LST (VIIRS)			
H-Pol Brightness Temperature (SMAP)			
VH-pol Backscatter Coefficient (Sentinel-1A)			
NDVI (VIIRS)	Soil Moisture	10.56	0.012
LST (VIIRS)			
H-Pol Brightness Temperature (SMAP)			
VV-Pol Backscatter Coefficient (Sentinel-1A)			
NDVI (VIIRS)	Soil Moisture	9.75	0.16
LST (VIIRS)			
H-Pol Brightness Temperature (SMAP)			
VH-pol Backscatter Coefficient (Sentinel-1A)			
VV-pol Backscatter Coefficient (Sentinel-1A)			

## Data Availability

Data can be made available upon request.

## Abbreviations

SMAP	Soil Moisture Active Passive
SFIM	Smoothing Filter-based Intensity Modulation
ANN	Artificial Neural Network
BG	Backus–Gilbert
VIIRS	Visible Infrared Imaging Radiometer Suite
SAR	Synthetic Aperture Radar
IW	Interferometric Wide
GRD	Ground Range Detected
MODIS	Moderate-resolution Imaging Spectroradiometer
AVHRR	Advanced Very-High-Resolution Radiometer
SeaWIFS	Sea-viewing Wide Field-of-view Sensor
OLS	Operational Linescan System
SDRs	Sensor Data Records
EDRs	Environmental Data Records
LST	Land Surface Temperature
VI	Vegetation Index
TOA-NDVI	Top of Atmosphere–Normalized Difference Vegetation Index
MLP	Multi-Layer Perceptron
